# Radiology artificial intelligence for prioritized imaging and diagnosis of lung cancer: qualitative interview analysis of stakeholder perspectives in Northern Ireland

**DOI:** 10.3389/fmed.2026.1759041

**Published:** 2026-04-02

**Authors:** Clare Rainey, Sonyia McFadden, Avneet Gill

**Affiliations:** 1 Discipline of Medical Imaging and Radiation Therapy, University College Cork, Cork, Ireland; 2 School of Health Sciences, Ulster University, Belfast, Ireland

**Keywords:** AI, artificial intelligence, lung cancer, lung cancer screening, patient centered care

## Abstract

Lung cancer is a leading cause of death internationally, with most cancers being diagnosed at an advanced stage. Initiatives, such as the Lung Cancer Policy Network promote best practice globally, such as development of screening programs, however, infrastructural issues such as staffing shortages may mean that changes to current practice may not be possible. Artificial intelligence (AI) has been proposed as a means to alleviate the pressure associated with additional imaging, triage and management. Principles of patient centered care should be adopted when considering any factor in healthcare. There is a dearth of literature on the patient and clinician perceptions of the impact of AI in the lung cancer pathway specifically, particularly in nations where this technology is being considered but not currently being utilized. This semi structured interview study recruited both patients and clinician volunteers who had responded to an initial survey on the same topic, resulting in seven members of the public and six clinicians. All participants reside in Northern Ireland, allowing for insight into a nation where AI had not yet been adopted in the lung cancer pathway. Interviews were coded and Braun and Clark’s recommendations for thematic analysis were followed, resulting in seven themes: 1. Person to person communication, 2. Use of AI in health – applications, 3. Validation, 4. Acceptability and variability of acceptance, 5. Education and training, 6. Patient consent AI in their care, 7. Workflow integration and infrastructural limitations. No themes were unique to either clinicians or public. Perception of the outlook for the future with AI in the lung cancer pathway was positive, and in many cased reported to be inevitable. Both clinicians and the members of the public highlighted the need for robust quality assurance to be in place. Opinions varied on the need for explicit patient consent to the use of AI in their pathway, with trust in the clinicians’ decision articulated by members of the public.

## 1 Introduction

Lung Cancer is the leading cause of death (20–25%) in both Wales, Northern Ireland (NI), with most cases diagnosed at a late stage leading to poor survival rates (12%) (1). This is a statistic mirrored internationally, with one in five cancer deaths attributed to lung cancer globally (2). The National Health Service (NHS) in the UK published the National Optimal Lung Cancer Pathway (NOLCP) in 2017 (updated in 2020) and describes steps to ensure timely access to evidence-based diagnosis and treatment for faster and early diagnosis and better patient outcomes (3). Similarly, the Lung Cancer Policy Network advocates and provides international support for earlier detection and improved care by influencing policy and promoting the use of low dose computed tomography (LDCT) for screening (4). However, it is evident that program provision varies greatly internationally and directly impacts global health disparity (5).

Due to increasing demand for imaging and workforce capacity constraints, NOLCP has not been completely achieved in UK, with only some locations in the UK where lung cancer screening is offered to high-risk patients (6), resulting in regional disparity in care. Artificial Intelligence (AI) is being proposed as a solution to transform and speed up the pathway for lung cancer patients from diagnosis to treatment (7). It is proposed that an AI-enabled Lung Cancer pathway could support:

1. Triage of urgent suspected cancer patients.
2. Early lung cancer diagnosis: management including malignancy risk.
3. Lung cancer patient management: monitoring progression over multiple follow up.

Alongside the introduction of AI into clinical practice, researchers across the international spectrum have examined service user perspective in relation to their care with a focus on providing person-centered care (PCC) (8–14). Studies indicate that relationship development between staff and service user, tailored information/knowledge provision and PCC are fundamental to positive perceptions in relation to care by service users (15). With effective communication and relationship development such a clear component in ensuring highly regarded PCC, some authors have queried the impact of AI on this dynamic (16).

This RAPID-LC project aimed to provide information on the patient and clinician perspectives on AI used in the lung cancer pathway in Northern Ireland (NI) as an example of a country where lung cancer screening has yet to be implemented. The unique situation of NI as a distinct geographical and political region means that the findings reported here may be generalizable to countries internationally where a screening program is yet to be established and where AI enabled lung cancer care is not yet realized. This may permit international development and implementation efficiencies. Eliciting the perception of the public and clinicians will ensure both PCC and the involvement of clinicians as key stakeholders in the design and potential implementation of AI assisted technology in the lung cancer pathway in NI, thereby promoting engagement and optimal implementation.

## 2 Method

This study aimed to investigate the opinions of key stakeholders on the use of AI for lung cancer diagnosis for both the screening and symptomatic populations, using Northern Ireland as an example population. Additionally, this study aimed to provide information on the ideal AI enabled pathway to allow for targeted feasibility studies and trials.

### 2.1 Recruitment

A call for participants was made via professional and general social media, specifically LinkedIn, X, Facebook and Instagram. Members of the public and clinical professionals who had responded to a survey on the same topic were asked to contact the researchers to take part in this phase of the study (Table 1). No incentives were offered to participants.

**Table 1:** Inclusion/exclusion criteria.

Inclusion criteria	Exclusion criteria
Adult members of the public who are receiving or who have received any form of medical imaging within the last 3 years	Have not received medical imaging in last 3 years
Clinicians involved in the lung cancer pathway in NI.	Clinicians not involved in lung cancer pathway in NI.
Resident in NI	Not resident in NI
At least 18 years old	Under 18 years old
Fluent English speaker	Non-fluent English speaker
Completed the survey on the same topic and volunteered to participate in this interview phase of the study	Did not complete the survey on the same topic

This study adopted a Constructivist Grounded Theory (CGT) approach (17), by basing prompts and questions of an earlier questionnaire phase of this study, therefore including the participants in the construction of the interview topics.

The recruitment of potential interviewees was directly informed by the constant comparison process of interview data gathered from those interviews completed and analyzed. This process of comparing data gathered identified themes emerging from the data in terms of their commonalities and differences. Once there were no new themes identified from analysis of the data gathered, the themes were described as ‘saturated’ with data collection and analysis running in tandem with each other to monitor reaching that point (17). Once themes were saturated, participant recruitment was terminated.

The semi-structured interviews (script available in Supplementary material 1) took place via Microsoft Teams and lasted approximately 40–60 min. The CGT methodology adopted accommodated the existence of multiple realities within the general population, allowing for in-depth analysis of service user experience in order to identify variations and similarities in those experiences to better understand them and provide a “unified theoretical explanation” (17). All interviews were video/audio-recorded and transcribed verbatim. Data was anonymized at the point of collection and only demographics in relation to the participants’ profession and experience with lung cancer were retained following transcription. Video data was used for clarity in relation to non-verbal cues. Once this information was captured in the transcription, and the video was deleted.

The semi-structured form of interviewing allowed the interviewer the opportunity to explore, in detail, the key themes not normally accorded in everyday conversation and allowed the interviewee to tell their ‘story’. In this way, the data collection process at this stage was interviewee-led.

### 2.2 Data analysis

Each interview was audio-recorded for later transcription. Adhering to the belief that ‘data analysis in grounded theory involves specific procedures which, when applied appropriately and with vigilance, will result in theory that is rigorous and well-grounded in the data (18). Whilst the study adopted a CGT approach, data analysis followed the steps of Braun and Clarke’s thematic analysis as the study was exploratory in nature, rather than the explicit construction of theory (19). The interviewers are active AI researchers and therefore the CGT approach was appropriate to allow them to use their knowledge to prompt the participants to elicit greater depth to the interview responses. The insight from the interviewer allowed for deeper investigation of the responses of the participants. Line by line coding, as per the CGT approach, was adopted to produce detailed data. However, thematic analysis was used in order to provide overarching themes to understand the perceptions of the barriers and facilitators to AI adoption in this area, without creation of theory. The following steps were undertaken in the data analysis of these interviews

1. Interview transcribed from digital recording
2. Transcription anonymized by the assignment of a labelling code based on the date of the recording
3. Line by line coding of the data
4. Comparison of the data with other data analyzed
5. Identification of emerging categories
6. Addition of definition and depth to categories
7. Making written records of coding and comparison to elaborate theory.

Coding of the data involved a process of close examination of textual data to derive meaning from it. Data analysis and data collection took place synchronously allowing the researchers to identify when data saturation had been achieved (17). As the coding process continued with subsequent interview data, the researcher evolved ideas and concepts from the data which contributed to the evolution of a conceptual model about the current perceptions of AI enabled diagnostic tools.

The data was cleaned by two researchers (CR/AG) respectively cleaning one participant group, i.e., public and clinician. This was to identify and rectify any hallucinations from the transcription software on Microsoft Teams^©^.

A two-stage coding process was employed: (i) initial coding and (ii) focused coding.

The data was then coded by the interviewers independently (AG/CR). No discrepancy was found; however, overall themes and codes were confirmed by a third researcher (SMF), using researcher triangulation to confirm meaning. Initial coding was conducted using line by line analysis. This method sticks closely to the original data, while identifying initial themes. A reciprocal approach was used to merge existing themes with those previously identified. Based on this grouping of themes, initial codes were produced which summarized raw data and began to condense and shape it through an abstraction process.

The researchers acknowledge their expertise in the field and that their individual viewpoints may have induced some bias. However, ‘field notes’ were kept by both researchers and the multi-step coding and reflexive process during data coding and analysis was conducted to limit unhelpful influence. The researchers agreed on all codes and themes and discussed any minor discrepancy in order to thoroughly question their own assumptions.

During the interview, clarity was confirmed by the researcher at the time of the data collection and recorded in the transcription. Two researchers conducted the interviews with one assuming the role of interviewer and the other collecting data in relation to the meaning of statement (i.e., keeping ‘field notes’ of non-verbal cues). Both researchers kept personal ‘memos’ during the interview.

All documents in relation to the study were retained and will continue to be retained for 10 years, as per the institutional research ethics requirements. These include:

Raw transcripts
Clean transcripts
Participant codes and associated background information (as included in Tables 2, 3)
Researchers’ ‘field notes’ (included as annotations on the cleaned transcripts)
Initial codes (included as Supplementary material 2)
Theme development (according to initial detailed coding)

**Table 2:** Participant professional background, experience with cancer (members of the public only) and AI experience.

Participant	Professional background	Cancer experience (public only)	AI experience
P1	Computer science—retired	Personal experience of cancer diagnosis – not lung	Not aware of having been used in care but aware of the technology and interested
P2	Management/organisation/administration	Close family experience of lung cancer	Not aware of having been used in care but aware of the technology and interested
P3	Medical – retired	Close family experience of lung cancer	Not aware of having been used in care but aware of the technology and interested
P4	Human resources	Personal experience of cancer diagnosis – not lung. Close family experience of lung cancer	Not aware of having been used in care but aware of the technology and interested
P5	Education / research	None	Very knowledgeable and uses AI as part of work
P6	Project manager	Family member – investigations for lung cancer	Aware of AI as part of work (non-medical applications)
P7	Accountant	Close family experience of cancer – not lung	Not aware of having been used in care but aware of the technology and interested
C1	Medical physics		Used clinically in previous work
C2	Nurse – retired		No experience
C3	Oncologist		Significant experience in previous employment for lung cancer specifically
C4	Radiographer		Aware but has not used clinically
C5	Nurse—academic		Aware and strong interest but has not used clinically
C6	Anaesthetist		Very aware, knowledgeable and strong interest but has not used clinically

**Table 3:** Themes and subthemes.

Themes	Subthemes
Person to person communication	Positive
	Negative
Use of AI in health – applications	Positivity about AI in health
	Concerns about AI in health
Validation	Human oversight needed
	Other means of validation and assurances
Acceptability and variability of acceptance	
Education and training	
Patient consent to AI used in care	Patient consent needed
	Patient consent NOT needed
Workflow integration and infrastructural limitations	

NVIVO^©^ was used to organize the data, codes and themes. The researchers then came together to form the final categories from their individually formed codes, allowing for the addition of depth and definition (Supplementary material 2).

#### 2.2.1 Ethical considerations

Informed consent was gained from all participants. Written consent was obtained prior to the commencement of the interview and confirmed verbally at the time of the interview, following an opportunity to ask questions. None of the participants withheld consent.

Ethical permission was sought and obtained from the Institute of Nursing and Health Research Ethics Committee at Ulster University (reference number FCNUR-24-114-A).

## 3 Results

Seven themes and eight subthemes emerged from the interview data of both the members of the public and clinicians (Table 4). Due to the similarity of the responses, the themes are presented collectively. No themes were unique to either clinicians or patients. The codes attributing to these themes are presented in full in Supplementary material 2.

**Table 4:** Public perception of the need for face-to-face communication with clinicians.

Quotation	Interviewee
*“You’re still wanting that sort of human connection. You’re still wanting, ultimately, a human being to maybe look at.”*	P2 (member of the public with experience of relative passing away from lung cancer)
*“…he said the best 18 months he’d had for a long time…because of engagement of people, not just, I mean, I mean friends and family, but not just that, but just engagement with all the various people who are involved in his care.”*	P3 (member of the public with experience of relative passing away from lung cancer)
“*…without a personal presence, whether it’s a consultant or whether it’s a specialist nurse, I think there is a need for a human interface in all of these things*…*we all use apps at different times for relaxation and stuff, and but if you have a problem, you need a person… If there’s anything there, I think it’s a human interface” (that is needed).*	P4 (member of the public with personal experience of cancer other than lung cancer)

Participant demographics are included in Tables 2, 3.

### 3.1 Themes

#### 3.1.1 Person to person communication

Overall, both clinicians and members of the public expressed positivity and desire for person-to-person communication and overwhelmingly feel that this is important to retain in the digital era. This sentiment is felt by both patients and clinicians, however in slightly different ways. Firstly, patients desire communication from a human when support is needed, such as delivery of bad news (Table 5).

**Table 5:** Clinicians’ perception of the need for face-to-face communication with patients.

Quotation	Interviewee
*“You went on a ward round and you were able to pick up the body language and the facial expression. And then you were able to go back to that patient and say, look, you know, I noticed you maybe were not sure of whatever. And then you were able to talk to him, you know, so.”*	C2 (experienced nurse)
*“Yep, really important to me. I think you can glean a lot about a patient just from seeing them for a few seconds. There’s lots of nonverbal stuff that inspection aspects of one’s examination, that it’s really: There’s no way to do it better than seeing the patient face to face, probably.”*	C3 (consultant oncologist)
*“…you spend so much time at a patient’s side, trying to establish that rapport and something that you get good at…”*	C4 (experienced radiographer)

Secondly, clinicians recognized the need for face-to-face communication for additional information, whether this be to Supplementary information already obtained from the patient or to indicate that extra support or explanation is needed (Table 6).

**Table 6:** Public perception of the impact of AI on interaction with the clinician.

Quotation	Interviewee
*“But I think it should also free up people to do the interpersonal stuff and that’s the bit about it. You know, people are so busy they do not have the time, and they do not have capacity…”*	P7 (member of the public with experience of a close family member with cancer)

Opinions varied on whether AI will have an impact on this. One member of the public felt that AI might be used to free up clinicians to spend more time with the patient (Table 7).

**Table 7:** Clinician perception of the impact of AI on communication with the patient.

Quotation	Interviewee
*“I think for me it would probably, I think, give you a bit more confidence in your interaction with patients…it would provide me with more of a confidence for the basis of my conversations with patients around diagnosis and investigations.”*	C5 (experienced nurse)
*“I’d be open minded to anything that speeds up the pathway for the patients and makes the communication between different members of the team a bit more useful…I’m aware of tools that you know can help with dictation, so they sit in the background of the room and they can convert the conversation that’s happened into a succinct letter.”*	C3 (consultant oncologist)

Clinicians feel that AI may have a positive impact on patient communication in some cases, such as helping the clinical staff to understand how to best communicate with the patient (in the case of the nurse participants) and may allow for better communication style (in the case of the doctors) (Table 8).

**Table 8:** Opinions of clinicians on the impact of AI on professional roles in the workplace.

Quotation	Interviewee
*“But this can make such a difference, and we have got to move on.”*	C2 (experienced nurse)
*I do not think there’s any question about AI not being present in the future. It’s there now already… it’s not a question of saying we’ll turn the clock back. That’s not feasible…Is there going to be AI? It’s there…”*	P3 (retired doctor with experience of close family member with lung cancer)
*“I would not want AI to replace something that’s good at the moment. You know what you can imagine in 50 years’ time that you know, funding for nurses has been cut and patients cannot get their own clinical nurse specialist.”*	C3 (consultant oncologist)
*“…the biggest fear I think of a radiographer in terms of AI is that one day scans and equipment will become automated and in that way there’ll be a reduction in patient care …And we are looking far into the future, but maybe they’ll not even be a radiographer there…my biggest fear is that one day it’ll be like a, you know, you know, like getting your passport photos…where there’s a curtain, it’ll just be automated voice and put your hand on the X.”*	C4 (experienced radiographer)
*“I think healthcare professionals initially are quite sceptical of AI and may be quite nervous of it. I think in in my kind of anecdotal conversations with people around it, it’s been that they have been it could, you know, there’s concerns around replacing their jobs.”*	C5 (experienced nurse)
*“… there will be people in radiology who have been doing chest X-rays for the last 40 years and they will go AI, ‘no, we do not trust that’, but they are maybe coming to the end of their careers…things have to move on. They just have got to move on.”*	C2 (experienced nurse)
*“I think there’ll be a lot of sceptics, we are semi nihilistic as a general tendency in Northern Ireland”*	C3 (consultant oncologist)
*“I think I do not know what I do not know what the evidence shows about this, but my feeling would be among younger staff. Potentially. Yes. I think among older, which is actually then the more experienced staff probably (not)…”*	C5 (experienced nurse)

#### 3.1.2 Applications of AI in health

Most opinions on the use of AI in health were positive and indeed, the use of AI is recognized as inevitable to support a stretched workforce. There is minimal fear of replacement of clinical staff with AI, however interestingly, this was not a concern raised by the public, but a sentiment present uniquely in the clinician population. Any concerns around job replacement centered on fear that AI might be used to reduce, rather than replace, services currently provided by humans and indication that there may be an age or experience-based reticence for adoption of AI in staff who are older or have been in their job for longer (Table 9).

**Table 9:** Perceptions of the potential for AI to speed up delivery in some areas yet cause “bottle necks” in others.

Quotation	Interviewee
*“…but that so it would really mean if you were employing something like AI and if it was working as everybody hopes, then further down the road from imaging then services need would need to be expanded upon because what you are going to have is you are going to have lots of nodules detected, increased cancer detection. So all these patients are coming out the other side…you obviously need then the people that are going to treat them or the bottleneck just be moved further down the road temporarily…”*	C4 (experienced radiographer)
*“There could be a bottleneck down the system…my initial thinking would be I would be wary about creating expectations that the system could not support”*	P1 (member of the public with experience of cancer and background in computer science)

Applications of AI were suggested which span the areas of treatment planning, triage, diagnosis and screening for both diagnosis and patient condition monitoring, indicating that many of those interviewed had some idea of the applications available currently. However, whilst some of the clinicians had used AI in the past (mainly for segmentation for radiotherapy planning), none were using it currently.

Interviewees had some mixed feelings on the impact of AI on the speed of processes – with responses indicating that they felt that AI would speed up diagnosis and time to treatment but that this would only be possible with a whole healthcare system approach. A unique sentiment present in the clinician responses indicates a need to recognize the potential for speed in one area to cause bottlenecks in others and that there may be a ‘whole system’ approach needed in relation to infrastructural insufficiencies currently (see also theme 7: workforce) (Table 10).

**Table 10:** Overall perceptions of the need for human oversight of AI.

Quotation	Interviewee
*“You know, I really think that AI should be there as a helper to us, not to replace anything. There’s nothing really urgently missing that AI needs to suddenly introduce as such…”*	C3 (consultant oncologist)
*“It would have could well be, before major decisions in terms or treatment of something that has been identified or even not identified for symptomatic people, there is human oversight.”*	P3 (retired doctor with experience of close family member with lung cancer)
*“Checking of the AI and its performance is that something that you would be comfortable being done that that, that has to be done by a human clinician oversight…and the important you think that ongoing quality assurance by a human clinician is important”*	P4 (member of the public with personal experience of cancer other than lung cancer)
*“I feel like I’d want both AI and a human looking at it…I’d be OK with them being read. I’d still like, just personally prefer if there’s also human eyes on it before there’s like a final decision made.”*	P5 (member of the public, no experience of the cancer pathway in NI)

#### 3.1.3 Validation

The need for robust and reliable validation came across very strongly from both clinicians and members of the public, although in slightly different ways. Both groups felt strongly that there was a need for the performance of the AI to be readily available and clear, however the clinicians were aware of the onus placed on them for the final decision. The members of the public indicated trust in the clinicians for the use of any tool in their care, as long as there was a degree of human oversight and confirmation.

There were two main subthemes emergent from the data:

Firstly, the need for human oversight, which was expressed in different ways, for instance, in relation to the final decision making. AI should be used as an assistive technology to the clinician and not replace the decision of the healthcare professional and human oversight of the ongoing quality assurance of the technology should be used (Table 11). Secondly, the interviewees were keen for other means of validation to be addressed. Many participants noted the requirement to know the performance of the model. This was common across both the clinician and the members of the public (Table 12).

**Table 11:** Overall perception on the need for transparency in AI performance and reliability.

Quotation	Interviewee
*“… I probably need to confirm whether my assumption is right. I’m imagining that before you introduce AI into a healthcare, it has to go through a similar level of testing as if you are introducing a new drug…that’s making sure that people are aware of that.”*	P7 member of the public with experience of close family member with cancer
*“I think people need to know are that … this AI has been has run 10,000 MRIs and has not got one wrong yet or has a 99.999% rate success rate. I think that’s important for people to know (that) it performs as well, if not better, than the human.”*	P4 member of the public with personal experience of cancer other than lung cancer
*“…you know the sensitivity and specificity and accuracy of nodule detection will probably be … well-advertised and people will be informed about that, but also given in the report”*	C4 experienced radiographer
*“However, I would probably want to know a bit more about the evidence behind it before they’ll be able to fully commit to that answer for patient for patients, … I mean I need to know how effective it was, and you know what the evidence base was.”*	C5 experienced nurse

**Table 12:** Need for transparent information on the training of AI tools.

Quotation	Interviewee
*“… there’s a huge importance to what data you are using to build these models. Because if you went into a hospital here in Northern Ireland and had somebody delineate (referring to segmentation) … and then you went to France and had it done there, you get a different result”*	C1 (medical physicist and medical student)
*“Generally, if you have a sufficient* var*iety of, you know, gold standard or ground truth, you can validate that and it’s a very useful technology from a diagnostic point of view.”*	C6, consultant anesthetist

One participant noted that in order to build trust with clinicians that any technology should be used on a “low risk” application first.

*“I think it’s the best way to build trust amongst people is to use it for something low risk to start with.” C1 (medical physicist and medical student).*

The *clinician* population noted the importance of the acknowledgement of the data used to train the model (Table 13).

**Table 13:** Clinician perception of technological acceptance.

Quotation	Interviewee
*“So you’ll have some people, I think, especially the people that have been working with AI and have been informed about it, they will probably be more accepting of it. I think as well there will be people who want to defend the boundaries of their profession and they probably will be less accepting of it*…”	C4 (experienced radiographer)
“… *I think as healthcare professionals, maybe we are quite suspicious of AI and maybe kind of intimidated by it too”*	C5 (experienced nurse)

#### 3.1.4 Acceptability and variability of acceptance

*Acceptability and variability of acceptance* was a common theme amongst clinicians and the public. The public were generally technologically inclined to accept AI use, though they lacked knowledge around what was currently present in clinical practice. There were a mix of responses from clinicians around technology acceptance, either a feeling of fear or intimidation relating to AI integration or honesty around knowing nothing about AI (Table 14).

**Table 14:** Overall perception on whether AI will be adopted.

Quotation	Interviewee
*“On it’s not a question of saying we’ll turn the clock back. That’s not feasible. The questions are, what’s it going to be like? How are we going to control how you know, all those questions are the ones to be addressed, not. Is there going to be AI? It’s there*.”	P3 (retired doctor with experience of close family member with lung cancer)
“*… I think it’s entirely reasonable that we adopt AI.*”	C3 consultant oncologist

When asked questions about the impact of AI use, one clinician replied “*Well, I think it’s so difficult for me because my knowledge of AI is so limited.”* C5 (experienced nurse).

When posed a follow-on question about their experience with AI, one clinician C2 (experienced nurse) stated “*Absolutely none whatsoever. I mean, when I first went into cardiology I would have seen, you know, stents being placed and that sort of thing. But no, there wasn’t anything about AI.”*

Whilst a member of the public replied “*Like, I’m not sure how much AI would be involved there I’m not up to speed on how much of the automation would be AI versus how much would just be …a type of statement.”* P5 (Member of public with no experience of cancer pathway in NI).

Participants gave the impression they felt it was all inevitable as AI already was in practice and all that could be done was to go through the motions (Table 15).

**Table 15:** Perceived education and training needs for clinicians.

Quotation	Interviewee
*“I suppose the staff just needs to do some workshops on AI. There needs to be, you know how it will be done. Who could do it…. just everything there is for the clinician and for them to understand, but also how to discuss it with patients? How to reaffirm what the consultant has the person talking about?”*	C2 (experienced nurse)
“*I think if people are using it and learning from it and seeing its strengths and weaknesses and then they can learn the best ways to integrate it into their workflow… But without training, without testing, without having a go, then they’ll not be able to use it. And I think like anything, we fear the most what we do not know about so often, whenever we are introduced to things and play with them and work for them a bit, then it breaks down the boundaries, does not it?”*	C4 (experienced radiographer)
*Yes, I think using trying to use the equipment or watching demonstrations and seeing where it can be used best using the platforms you know having a go to see how user friendly…”*	C4 (experienced radiographer)
“I *think what would be probably useful is to hear from people/ from clinicians who have successfully implemented AI and found a huge benefit from it. You could give them a lecture about AI that’s probably not going to change their mind. …. obviously it is important for them to understand what it is and how it’s trained and what’s involved and stuff like that. But I think the more effective option would be to have somebody who has used it in their centre and it has benefited them and benefited the patients.*”	C1 (medical physicist and medical student)
“[training] … d*epends on what the involvement of nurses would be in the process. But I think on any level that training would be really helpful*.”	C5 (experienced nurse)
“*There will need to be radiologists…like consultants or people who have been highly trained to be totally specialized in AI.”*	C1 (medical physicist and medical student)

As mentioned in the *Applications of AI in health* theme, members of the public were positive about the use of AI in practice, though one clinician mentioned the impact of patients getting more training on the use of AI to further public acceptance.

“*I think maybe in terms of patient feedback, you might get some, some people who maybe would not be that happy that AI is being used, you’ll also get other patients who will be absolutely thrilled that AI is being used in their care. That we are moving forward to the future and everything seems very futuristic. That we are able to use technology they’ll be thrilled that you know they have. If people who maybe have a deeper understanding of what AI is and what it does and how exactly it was used in their care, might even appreciate how this meant that there was more time to have better treatment in, you know, receive more time for staff to deliver better treatment in other area…” C1 (medical physicist and medical student).*

Generational differences in acceptance of AI were mentioned by both members of the public and clinicians, with the main sentiment being that the older generations may be slower to accept AI than the younger generation.

*“…there will be people in radiology who have been doing chest X-rays for the last 40 years and they will go AI- No, we do not trust that, but they are maybe coming to the end of their careers and the sooner they go the better. They retire the better because things have to move on. They just have got to move on.”* C2 (experienced nurse).

When asked about potential barriers to AI acceptability, C4 (experienced radiographer) stated *“I did not see any barriers, but I suppose you know you’ll probably have the old-school.… reporters who probably will not want to use it as much as the younger people.”*

When asked about acceptance amongst clinicians C5 (experienced nurse) stated “*I think I do not know what I do not know what the evidence shows about this, but my feeling would be [acceptance] among younger staff. Potentially. Yes. I think among older, which is actually then the more experienced staff probably.”* “*…I think my level of experience kind of bridges the two that you know the staff that are younger than me or newer than me are much more … comfortable with technology in every form. And the staff that are more experienced or more resistant…”*

#### 3.1.5 Education and training

*Education and training* were raised by the majority of clinicians and members of the public as necessary alongside, and prior to, AI integration within clinical practice. Many of these sentiments were linked closely to acceptance of technology. However, there was confusion regarding what type of training or education would be appropriate for the different demographics (Table 16).

**Table 16:** Education for the public.

Quotation	Interviewee
“*I would not have thought of major training program was needed…” “I would have thought awareness and in today’s world of apps and tablets, many people are very quick at picking things up.”*	P1(member of the public with experience of cancer and background in computer science)
…awareness of AI should begin in early education *“Like I do not want to say it’s too late for outside of school. But I would say like focusing on early education on like primary and secondary education. Almost reforming the curriculum like there’s an opportunity there to, to really show people. And embed AI in the curriculum, but to really start children thinking this is how to think about this. You know think that’s going to be around now? Rather than having to like rethink about it after they are done with school.”*	P5 (member of the public, no experience of the cancer pathway in NI)
“*That communication strategy around that is about using the social media platforms, whatever they may be, and using some of those influencers or creating the really specialist people. But as some sort of personality. Where they have a sense of being trusted… It’s people like that who need to get out to the social media platforms and not quite ignore the printed media or even mainstream television. But through that process, that influence the process.* [AI integration]”	P4 (Member of the public with experience of cancer other than lung cancer)

Additionally, there was mention of an AI specialized clinician from one of the members of the public P1 [*member of the public with experience of cancer and background in education (computer science)*], suggested that in place of training, awareness was required to be raised to the public (Table 17).

**Table 17:** Patients consent to the use of AI and their data.

Quotation	Interviewee
*“So far as AI being used, I think they should be told about it. I think my mind would be. Then what happens if they say no? Like is there an alternative? Like say it’s something that’s embedded into a process. Do you know?”*	C5 (experienced nurse)
*“… I would not like us to steer clear of discussing and telling people of the use of AI in their assessment.”*	P3 (retired doctor with experience of close family member with lung cancer)

#### 3.1.6 Patient consent for AI in care/patient do not consent for AI in care

An interesting sentiment that arose from speaking to participants related to patients consenting prior to AI use in their care. Varied responses from the public and clinicians were noted (Table 17).

One member of the public noted that consenting to AI could be necessary before it is trained and validated “*And initially I think while we are building confidence and testing it and doing all of those people should probably be given a choice. But I think after that, once it’s tried and tested, if it that’s the first point of call and it’s proven then no.”* P7 (*member of the public with experience of close family member with cancer*).

One of the clinicians suggested it was necessary for patients to consent to AI use in a simple way *“Well, I think at the end of the day, what needs to happen is it needs to, it needs you know there needs to be a survey done after the AI is you know if you are doing AI, think there needs to be a survey done and a very simple tick box and then you know obviously you if someone’s going to get AI you would produce a booklet and then some of these comments could be in it.*” C2 (experienced nurse).

However, some clinicians suggested that there was no need for patients to know if AI was used in their care.

*“So do I think a patient would need informed at every stage and should they accept that? I do not think so, I do not. I do not know why I think that if they can prove that there is a real high sensitivity and if that can be proven within the field of medicine… and that it’s without a doubt, then a part of me feels that patients, some patients, irrational fear of computers making decisions might lean them towards making the wrong decision…. So I’ll say no.”* C4 (experienced radiographer).

One member of the public trusted the use of AI alongside a clinician and saw no reason to consent to its use (P6-no prior experience of lung cancer), “*No, and I thought, I think that’s it. If it’s been used as a tool like anything else, I would almost kind of see it as the equivalent of them consulting with a colleague. And as long as the final decision and the final kind of review of it was to know with qualified radiographer or consultant or something …”*.

For patients, there was a feeling throughout the interviews that related to personality driven decision making, i.e., if the patient’s personality was naturally inquisitive, then it could be that they would want to know about what was taking place in their care.

“*I’ve been in wards for* var*ious reasons and beside me have been medical staff. Whether it’s a change in trend or what or not are very good at telling exactly what they are going to do. I like that. I like, try and understand what the procedure is, but I’m aware that there are many folks who do not like that*.” P1 (*member of the public with experience of cancer and background in computer science*).

#### 3.1.7 Workflow integration and infrastructural limitations

Finally, *workflow integration and infrastructural limitations* were noted by participants as one of the limiting factors affecting the implementation of AI within clinical practice. All relevant quotes are presented here rather than in a table. Clinicians noted the nuances attached to implementing AI within radiology relating to the *Validation* theme,

“*I think it takes a bit of work to get it established in the hospital and there’s obviously time to sort of, you know, tender and work out which auto AI solution is going to be best for each hospital or each trust*…” C3 (consultant oncologist).

C6 (*consultant anesthetist*) noted that there are cognitive processes that need to be considered alongside AI implementation “*I think there’s a lot of what’s like workflow, cognitive engineering, cognitive ergonomics, the way these things are designed to fit with thought processes and the known. I mean we already know if there’s, you know that radiology, what you tend to zero in on the abnormality and forget about everything else. So, I think in radiology there’s a lot of cognitive science that we now have to explore to see how we make this safe because a simple hypothetical right solution needs to be proven to be safe at scale. So now we can we know the model will have a certain performance*.”

There was also an understanding from clinicians that implementation of AI would have a direct effect on other services within the hospital setting.

“…*so, it would really mean if you were employing something like AI and if it was working as everybody hopes, then further down the road from imaging then service need would need to be expanded upon*” C4 (experienced radiographer).

This same sentiment was suggested by a member of the public, though with the caveat that this could have negative implications for raising expectations on the timescale of care delivered. When speaking about their own experiences about delays in the emergency department, P1 (*member of the public with experience of cancer and background in computer science*) noted *“But AI would raise expectations. There could be a bottleneck down the system and so… My initial thinking would be I would be wary about creating expectations that the system could not support.”* This relates heavily to the *AI Applications in Health* and the pragmatism of having AI technology in widespread use. P1 (*member of the public with experience of cancer and background in computer science*) further noted that they “*Sometimes sense some people really like to be ill. Today It [AI] could fuel that sense of… that sense of needing treatment and of needing help. And so, if they had a tablet [electronic], they could assess themselves, they could be finding all things wrong with them*.”

## 4 Discussion

Overall, the perception of the use of AI in the lung cancer pathway was positive. Cognizance of the need for validation, education and some workflow issues in relation to the procurement and ongoing quality assurance.

### 4.1 Person-to-person communication

Participants clearly articulated the need for person-to-person communication, in particular in the “digital age”. This was felt to be particularly important when delivering bad news. Derevianko et al. (20) report the findings of a systematic review into the use of AI in cancer diagnoses and the impact of this on patient confidence in AI and found that patient trust and communication is negatively associated with the use of AI. This was found to differ depending on certain demographic characteristics, such as level of education. This is concerning as this may lead to further disparity in care for certain patient groups.

Clinicians also noted that communication with patients was not only to do with oral communication, but rather, the holistic information gained from non-verbal cues. Around how the patient is feeling or their level of understanding. However, the impact of AI on communication was not felt to be strongly positive or negative. There was some indication that AI may free up time to spend with patients and make communication more efficient and allow frontline staff to communicate accurately with patients. This is supported by findings from a review by Sauerbrei et al., who proposed that the impact of AI could be double edged – it could free up healthcare professionals to spend more time with their patients or allow for time to be created to fill with additional work for the clinician (21).

### 4.2 Applications

There is still fear regarding the replacement of staff or roles with AI, but interestingly, this was a unique sentiment in the clinician population, who articulated concerns related to the reduction in service provision. Clinicians felt that there was a risk in AI replacing human-facing tasks that humans do well, such as positioning for scans and providing supportive care to the patient. Whilst there was positivity about the impact of AI generally, there was concern about variation of adoption in the older, more experienced population and that this may hinder adoption.

As expected, of the clinicians interviewed, none were using AI currently. This is a concern as exposure to AI systems is needed to be able to champion their use and input into the specific needs of the care setting. This may be due to myriad reasons, such as procurement issues and infrastructural barriers. With many applications available for AI in lung cancer, from histology classification, treatment efficacy prediction, radiology-based diagnosis and reconstruction of images (allowing for dose efficiencies for screening) (22–24), education and awareness of AI used in lung cancer applications should be prioritized to allow clinicians and patients to gain insight into the advantages of the use if AI in this setting.

Interestingly, there were mixed responses on the impact of AI for reduction in time-to-scan, speed of diagnosis and time-to-treatment. Clinicians, having knowledge of the current workflows in the health care setting, issued concerns about the speeding up of one aspect of the patient journey. They noted that this may result in bottlenecks in other places along the workflow.

Other predictive applications such as the personalization of screening programs (22) and prognosis and treatment response predication (23) were not mentioned. These AI applications demonstrate areas where AI is providing new information to support clinicians delivering personalized patient care and treatment planning but are, perhaps, still in the relatively early stages of development and implementation.

### 4.3 Validation

Whilst validation was mentioned by both members of the public and clinicians, the sentiment was different. The clinicians were aware of the responsibility for the final decision and the use of AI in the patient’s care, and the members of the public were content to place their trust in the clinician to use what they felt appropriate, provided there has been human clinician oversight.

Both felt that AI should be used as a second opinion and as an assistive technology and that there should be human-led quality assurance of any tool used in patient care. This is supported by literature suggesting that Acceptance Testing (AT) at the point of implementation followed by robust Quality Assurance (QA) and Quality Control will support trust and prevent undesirable evolution of the performance of the technology (25). Clinical AI champions have been suggested to ensure responsible use of AI and may encourage adoption and efficient implementation (26). Trivedi and colleagues provide an example of the establishment of a Radiology AI Council for oversight of the implementation and roll-out of AI in a large clinical center (27). This approach is supported by Kim et al., who describe how a similar group provide realistic expectations from the AI applications in use in their clinical center in the Netherlands (28).

Both clinicians and members of the public expressed a desire to have an awareness of the performance of the model being used. The clinician participants expressed knowledge of the need to have scrutiny of the training dataset and expressed an awareness of the lack of generalizability of the datasets used for training.

### 4.4 Acceptability and variability of acceptance

There was a great variability in the responses around acceptance. This reflects the literature where acceptability of technology varies, however both groups noted the inevitability of AI being used in clinical practice and subsequent need for professionals to be prepared to engage with AI. There was some mention of a reticence of the more experienced or older clinicians to adopt AI and that younger staff may be more comfortable with AI in their day-to-day lives and therefore may be more prepared to use AI in their work. Lambert et al., report findings from a review of the literature regarding health professionals’ acceptance of AI and report that there is no consensus in the literature around the impact of age on the acceptance of AI (29). Out of the three papers where age was investigated, two of the included studies indicated that younger people may trust AI more readily, however other included papers showed no correlation with age in willingness to interact with AI.

The impact of AI in relation to the level of experience is clearer and has been reported in a number of studies, showing that those with less experience may be susceptible to adverse interaction issues associated with the use of AI, such as automation bias (30, 31). Dratsch et al. report findings from a recent study investigating the impact of AI feedback on radiologists interpreting mammograms and found that whilst all participants were susceptible to automation bias, the inexperienced clinicians were more likely to over-diagnose (i.e., provide a higher BIRADS classification) based on AI feedback, compared to both moderately (*p *= 0.044) and very experienced radiologists (*p *= 0.009) (32). This effect is also found in the radiography profession. Rainey et al., report that student radiographers were more likely than more experienced professionals to follow the advice of an AI decision support tool when providing diagnosis for fractures on plain radiographs of the musculoskeletal system, therefore demonstrating automation bias tendencies in this population (30).

### 4.5 Education and training

The members of the public and clinicians both articulated the need for training in AI and related this to implementation success. This has been echoed in many avenues of healthcare, where current education provision has been found lacking (33–35). Education has been linked to responsible use of AI in the healthcare setting and may be a key factor in the appropriate use of AI (35–38). A recent American survey by Stogiannos et al., found that many educators (59.1% of 373 responses) recognized the importance of teaching AI to pre-registration radiographers, many lacked the knowledge to do so, indicating that training is needed for those who clinicians and the public may look to when providing educational support (34).

The format of education for the public and clinicians is not agreed between the two groups, or within the groups themselves. Some suggestions suggest that the public should have an accessible format which would demystify AI and debunk some inaccurate representations in the media.

Some of the clinical staff indicated that practical workshops would be desirable and may fit into their busy work and that if they will be using it there is a need to engage with it in a practical way in order to become accustomed to the usability of it. This is supported in a survey of radiographers by Rainey and colleagues in the UK, indicating that the format of education may need to fit with the clinicians’ working pattern, and suggest that CPD courses and e-learning/webinars may be preferable. Similar preferences are reported in nursing, by El Arab et al. (33), who emphasize the need for CPD activities and emphasize the importance of case studies for learning.

Responses from this study included a suggestion that a clinician would benefit from hearing from a colleague who is using the technology. This has been suggested in the form of clinical ‘AI champions’ and has been supported by initiatives such as the NHS AI Champions Initiative in collaboration with the Rai UK Health and Social Care Working Group (39). The impact of AI Champions has been reported in implementation studies, such as the study on barriers and enablers to successful adoption of AI in the Netherlands by Strohm et al., where AI champions were found to be a key factor in the successful implementation of AI.

For the members of the public, the optimal approach for AI education would be most advantageous through channels already available, such as social media, and beginning in school. However, careful scrutiny should be made of any online material as highlighted in a systematic review paper by Thapliyal et al. (51) where the quality of Social Media Health Information (SMHI) was found to be low and that the public articulated concerns about the veracity of the information. The publication of this information should be presented from reliable sources, such as professional bodies and AI experts (such as publications from the Society and College of Radiographers AI advisory board) and the public should be aware of the source of this information to ensure trust. This is supported by Anawade et al., who emphasize the ‘transformative potential’ (p.1) of social media but caution that there may be issues with the spreading of misinformation on such platforms and propose that further research and policy development is needed to use social media in the healthcare setting (40).

### 4.6 Patient consent for AI in care

There were mixed feelings about the need for explicit consent from patients for the use of AI in their care. Some suggestions were made that after the initial testing; patients may not need to be informed when AI is used. Similar to the sentiments expressed earlier regarding the desire for clinician responsibility for validation of AI models used in care, the interviewees felt that the responsibility for the use of AI in patient care should rest with this clinician. This is on the understanding that the clinician has deemed it fit for purpose.

The information available for patients on the NHS Transformation Executive gives patients information on how AI may be used in their care and how this consent is implied and not explicit (41). It is explained how patient data may be used to train the AI models of NHS collaborators. As a minimum, patients may need to have some indication given as to how their data will be processed by any AI system used in their care. This will allow them to have control over their personal health information and main trust in the patient-clinician relationship (42). In a systematic review paper by Moulaei et al., complexities in the obtaining of patient permission and inadequacies in the gaining informed consent were noted, resulting in recommendation for guidelines to assist clinicals and policymakers (43). Whilst obtaining consent for the use of AI in patient care is pervasive in the literature, Pruski, debates that from a legal perspective, the current legal frameworks in relation to both patient consent and GDPR are sufficient and that in the future, it may be impossible to guarantee the lack of use of AI in an individual patient’s care (44). In support of the findings from this study, Pruski advocates for robust evaluation and regulation of AI technologies by the clinical users, who use the AI systems for the benefit of the patient. This raises important ethical issues such as that of fully informed consent. This is a particular issue with the use of ‘black box’ AI models, where the functionality of the model may not but fully appreciated by the patient, nor indeed the clinician. The EU AI Act (45) is arguably the first and most comprehensive AI law but is focused on the development and deployment of AI tools, rather than the impact on the patient as the end user in terms of consent and use of personal data. The General Data Protection Act (46) can partly address this, although this Act does not fully address the complexities associated with the use of modern AI. With significant variation in legislation internationally, care should be taken by each user to ensure compliance with regulation in their country.

Supporting this, there was indication in the responses that the clinician should be able to explain the AI model and decision, if required by the patient. This may be an issue where not all clinicians are fully confident in AI. A recent study by Rainey et al., found that ‘reporting radiographers’ (i.e., radiographers who have obtained additional postgraduate qualification and experience to provide a formal written report) understood AI and had some knowledge, however, were not confident articulating this to patients and their careers and patients (47). This justification of the use of AI in patient care may become more important in the future when patients become more aware of advanced technologies in medicine and are more knowledgeable to engage in discussions about its use.

Technology acceptance frameworks such as the Technology Acceptance Model (TAM) (48), the Unified Theory of Acceptance and Use of Technology (UTAUT) (49) and the BEhavior and Acceptance fRamework (BEAR) (50) are useful means to help understand the relationship of the user with the technology. These can assist with the design of the technology, the construction of a technology-enables culture and ensure and measure how the technology is being used. Each model is slightly different and should be chosen based on the technology used and the research question posed. For instance, the BEAR framework is specifically designed to investigate the factors impacting whether clinical decision support systems are effectively used and adopted by clinicians, whereas the UTAUT framework is more general and not restricted to clinical technologies, but helps understand the motivating factors to affective use of technology. It may therefore be useful to utilize a combination of these frameworks to understand the acceptance of AI and emerging technologies in cases where a sufficiently specific framework does not exist.

### 4.7 Limitations of the study

This study was conducted in NI only and the results may not be fully generalizable to other nations in the UK or beyond. Participants in these studies generally had attained a third level education. Furthermore, some of the ‘members of the public’ in the interviews had previous experience in healthcare, with one a retired medical doctor and another, a senior managerial position in the NHS. Further study should focus on obtaining the perspectives of a range of participants with variations in demographic information. There was underrepresentation from those who have been involved personally in the lung cancer pathway in NI, however there were two out of the seven members of the public who had close relatives with lung cancer and a further three who had experience with other cancer pathways, either personally (*n* = 2) or first degree relative (*n* = 1). Further research involving those who have been engaged in the current lung cancer pathway may provide further insight.

In the clinician group there were only two medical doctors (one participant was a medical student, with previous career in medical physics in oncology). One participant was a radiographer and there were two experienced nurses. Whilst this professional mix may provide a useful overview of clinical perspective the absence of respiratory physician involvement is noted and would be useful in subsequent research.

Due to the time constraints with this study these issues were not able to be remedied. Despite multiple calls for participants, recruitment was difficult, resulting in a relatively small sample size. However, analysis was conducted using the recommendations by Braun and Clarke (19), who propose that the depth of the data is more important than a specific number. The participants are applicable to the research question and each interview resulted in a large volume of data. The codes were developed during data collections and by the final interview, in both groups, no new codes emerged.

Ethical approval for this study was granted based on recruitment taking place via online promotion, i.e., social media channels. This was to allow the study to begin as soon as possible. This may have contributed to the limitations noted in the previous paragraph. We found that despite ‘tagging’ cancer charities, the post was not reshared. Further studies should obtain ethical approval to specifically target populations who have engaged with lung cancer services or to recruit through NHS sites. Furthermore, there are issues with the use of social media for participants recruitment, namely, the potential of selection bias towards those in the population who are engaged with these platforms and those who are digitally literate. A recruitment strategy with wider scope should be adopted in future studies in this area.

The interviewees were recruited through expressions of interest within a previous survey on the same topic. This may have resulted in selection bias and indeed, in the background information (included in Table 1), many of the respondents have had exposure to cancer service and are interested in AI. Additionally, the participants were presented with an example of an AI platform from one vendor. This was to allow a more meaningful discussion around the topic and the possibilities of what AI might be capable of in this setting and no questions were asked about the platform itself. However, as all AI platforms are not identical, it is possible that exposure to this specific platform may have impacted on the findings although due to the relative similarity of the offering available on the market, this is not felt to be the case. Further research might consider presenting a number of platforms from different vendors.

## 5 Conclusion

Overall, the patient and clinician populations in this study indicated that the use of AI in the lung cancer pathway is positive, with some indicating that it is inevitable. Human oversight is preferred by many, however both clinicians and patients are more open to the potential for the use of more autonomous forms of AI, particularly in the asymptomatic/screening population. This comes with the caveat that the AI system is well validated, has demonstrable positive impact on patients (mostly in terms of speed) and subject to rigorous QA by human experts. The limitations of AI are recognized by both groups and there was almost unanimous agreement that AI should be used as a ‘second opinion’ and that humans should always have the ‘final say’ on a diagnosis.

There is some debate on the need for explicit patient consent, and this is an area that may require further investigation. Investment in patient and clinician education should be a priority before implementation of any AI system and the co-design of this should be an area for ongoing research.

## 6 Recommendations

- Patients and clinicians highlight the importance of person-to-person communication, and many have had excellent experiences in hospital settings. Measures should be in place to ensure that this will not be impacted by AI integration.
- Delays in time to scan and referral were noted – any dissatisfaction in the current system were related to workflow issues, rather than staff interaction. AI may have a place in workflow automation, and this may be a priority in AI implementation in lung cancer pathway.
- Patients and clinicians both express a desire to be informed of the performance of any AI model used in clinical practice. Developers should provide transparent data in relation to the performance of their AI solution and clinicians using the system (or appointed ‘AI champions’) should be able to interrogate performance metrics. Training data and transparency of AI technology is important to both clinicians and members of the public to ensure trust in the technology.
- There were mixed opinions on the need for patients to be informed about the use of AI in their care, from both clinicians and public indicating the need for further research in this area. In the interim, this should be considered on a local level to ensure that due procedure is being followed in relation to legal and ethical frameworks.
- Acceptability of AI amongst clinicians and the public could be propelled by training and education. Particularly, case-based training/practical training is desired by clinicians alongside AI integration. Training in AI is also desired by the general public. Social media has been proposed as means to do this, however further research should be conducted into its use in this population and in this subject, which is already fraught with misinformation and media hype. Formation of specialist groups of clinicians in professional bodies, working in tandem with AI system experts should provide accessible information to patients to meet this need. Further co-design of education tools should be considered to meet this objective.

## Data Availability

The raw data supporting the conclusions of this article will be made available by the authors, without undue reservation.
