# Supplementary material for: Radiology artificial intelligence for prioritized imaging and diagnosis of lung cancer: qualitative interview analysis of stakeholder perspectives in Northern Ireland
Source: Front Med (Lausanne). 2026 Apr 2;13:1759041. doi: 10.3389/fmed.2026.1759041 (PMC13083012; doi:10.3389/fmed.2026.1759041)
Supplement: Supplementary file 1 [file Table_1.docx]

Supplementary material 1 – interview script

HOUSEKEEPING/INTRO

Introduce self

My name is ***. Job title and place of work

I am co-investigator in this study. My role will be to gather and analyses this data and the data from the survey you have already taken. I am an experienced researcher and will ensure good research practices throughout in my role as a researcher and academic.

Lung cancer is a leading cause of death in NI. Most cases are diagnosed late, with poor prognosis. With current pressures in the health service and significant advancements in clinically available AI technologies, alternative ways of clinical management, from diagnosis to treatment planning and access to other services through patient journey planning are needed. This study focusses on AI used in diagnosis for lung cancer in Northern Ireland specifically. Your perceptions will be used to provide focus for further feasibility studies. Qure is only one company who provide AI diagnostic assistance for chest imaging however we partner with then specifically in this study, funded by the Small Business Research Initiative (SBRI).

This study aims to

1. To investigate the symptomatic/asymptomatic *service users’/general population* perception of the use of AI models in the lung cancer pathway in NI.
2. To gain *service users’/general population* understanding of the level of trust, acceptability and appropriateness of AI as used in screening and diagnosis of lung cancer in NI.
3. To provide *holistic stakeholder* (clinician/service user/general population) recommendation for the ideal AI enabled lung cancer pathway for the NI demographic, to allow information for further data-driven prospective clinical trials.

Instructions for Teams – if drop out, please try and re-access the link

Please speak as clearly as possible

The data will be fully anonymised

This is a safe space – the data will not be attributed to them individually to them in any way

The video will be used for non-verbal cues only – the video will be deleted after

Pseudo-anonymised until the point of analysis – assigned a numerical code (number e.g., P1, P2, C1, C2… and date)

The only people that will access this data are the research team – SMcF, AG and CR

No data will be shared further than this or with the AI company, or the funder. You have the right to withdraw up to the point to analysis, after this, due to the anon nature of the data at this point, they cannot be traced for redaction.

Appendix 4 a-patients

Stakeholders’ perspectives of using AI to aid lung cancer diagnosis

**Interview Schedule:**

Introduction

What is your background/your previous job role?

Tell me about your involvement with medical imaging for diagnosis....

1. What service/trust have you been involved with?

2. How long have you been involved with that service?

3. Do you have an underlying medical condition?

4. What do you remember most about it? (What was positive? Not so positive?)

Experiences of imaging, medical consultation

5. Thinking about your examination/diagnosis, can you tell me about that process?

6. Who has been involved in that process with you? (Staff, family member, carer, other professional)

7. Did you have to wait long for the examination/diagnosis?

Engagement with Staff

7. How do you engage with staff? Which staff do you engage with?

8. How important is this engagement for you?

9. What has been the impact of your engagement experience?

10. Would you change anything about this engagement?

Communication with staff

12. How do staff communicate with you?

13. What do staff communicate with you about?

14. How important is this communication to you and why?

15. Would you change anything about this communication?

***15/20 minutes***

Impact of AI

16. Do you think the use of AI assistance in imaging/diagnosis would impact this experience?

17. Are you aware/would you like to be aware of any AI assistance in imaging/diagnosis?

18 Would you be happy for AI assistance in your imaging/diagnosis? What if is sped up your imaging/diagnosis/treatment? Do you perceive any problems with AI assisted imaging/diagnosis/treatment? and why?

Perceptions of AI in diagnosis

20. What is your perception of the use of AI models in the lung cancer pathway in NI.

21. Thinking about the possibility of AI generated diagnosis – what are your perceptions on trust, acceptability and appropriateness of AI used in screening and diagnosis of lung cancer in NI? is there any advice that you would give?

22. Do you think AI assisted technology will impact time to scan/time to diagnosis/time to treatment.

23. Is there any training or education on AI assisted technology that you feel would be of use to patients?

Appendix 4 b- Clinicians

Stakeholders’ perspectives of using AI to aid lung cancer diagnosis

**Interview Schedule:**

Introduction

Tell me about your involvement with medical imaging for diagnosis....

1. What is your background and what service/trust have you been involved with?

2. How long have you been involved with that service?

3. Have you any experience working with AI assisted technology

Communication with colleagues (can you walk us through your day-to-day activities and which colleagues you would interact with)

12. How do colleagues communicate with you?

13. What do colleagues with you about?

14. How important is this communication to you and why?

15. Do you feel use of AI assisted technology may affect this communication?

Engagement with patients (and the patients you would interact with)

7. How do you engage with patients?

8. How important is this engagement for you?

9. What has been the impact of your engagement experience?

10. Do you feel use of AI assisted technology may affect this engagement?

Experiences of imaging, medical consultation

5. Thinking about your patient diagnosis, can you tell me about that process?

6. Who has been involved in that process with you? Would you be agreeable to AI assisted technology triaging your patients when scheduling for imaging/medical consultation/diagnosis?

7. Would you be agreeable to AI assisted technology double reporting alongside you?

***15/20 minutes***

Service User Involvement in Planning/Joint Goal setting for their treatment

17. Do you feel that patients should make a contribution to the decision-making about their care? In what way?

18. Are patients able to give any feedback on the care you deliver?

19. Do you feel that this contribution/feedback is important? Do you think the use of AI assisted technology would impact this?

Perceptions of AI in diagnosis

20. What are your perceptions of the features of the AI platforms in relation to system usability (including system usability scoring (SUS) and identification of barriers and enablers).

21. What are the optimal features of any AI model used on the symptomatic and asymptomatic patients’ lung cancer pathway in NI.

22. Do you think AI assisted technology will impact time to scan/time to diagnosis/time to treatment.

23. Thinking about the possibility of AI generated diagnosis – what are your perceptions on trust, acceptability and appropriateness of AI used in screening and diagnosis of lung cancer in NI.? is there any advice that you would give?

21. Is there any training that you feel would be of use to the staff?
