# Supplementary material for: Radiology artificial intelligence for prioritized imaging and diagnosis of lung cancer: qualitative interview analysis of stakeholder perspectives in Northern Ireland
Source: Front Med (Lausanne). 2026 Apr 2;13:1759041. doi: 10.3389/fmed.2026.1759041 (PMC13083012; doi:10.3389/fmed.2026.1759041)
Supplement: Supplementary file 2 [file Table_2.docx]

**Supplementary material 2 – codes and associated themes**

**THEMES:**

| **Themes** | **Person to person communication** | | **Use of AI in health – applications** | | **Validation** | | **Acceptability and variability of acceptance** | **Education and training** | **Patient consent AI in their care** | | **Workflow integration and infrastructural limitations** |
| --- | --- | --- | --- | --- | --- | --- | --- | --- | --- | --- | --- |
| **Subthemes** | **Positive** | **Negative** | **Positivity about AI in health** | **Concerns about AI in health** | **Human oversight needed** | **Other means of validation and assurances** |  |  | **Pt need to Conseco** | **Pt do NOT need to consent** |  |
| **CODES** | AI will IMPROVE communication (3,7) | AI DETRIMENTAL for communication with patients (2,3) | AI for decision or diagnosis prompting (5,6) | Clinicians concerned re AI taking jobs (3,4) | AI as an ASSISTANT (3,4) | AI performance info needed (6,12) | Acknowledgement of cognitive biases with AI (1,1) | No need for training (2,2) | Liking being informed about care (patient) (1,1) | NO need for pt consent (4,6) | Acknowledgement of limitations of current technology (2,3) |
|  | AI will NOT change person_person communication (2,3) | Ethical issues with AI (1,1) | Positivity about AI generally (4,5) | AI may NOT speed up diagnosis (3,3) | AI as assistive technology or second opinion (2, 5) | AI for something low risk to build trust (1,1) | Autonomous AI = ok (2,3) | Early years education in AI (1,1) | Public should know if AI used in care (5,8) | Do not need to know what tech is being used in care (patient) (2,2) | Moving too fast (intro of AI) (1,1) |
|  | Communication with patients  (2,2) | Face to face communication essential for patients (2,3) | Convenience of AI. (3,1) | AI in diagnosis (2,9) (neutral) | sensitivity of AI  (6, 17) (neutral perspective) | AI as second opinion (7, 16) | Lack of full trust in AI from clinicians(1,1) | responsibility  (1,1) | Patients should be told about AI use  (4,7) | Patients should not be informed of AI use  (4,5) | Red tape/infrastructural limitations (2,3) |
|  | AI gives CONFIDENCE in diagnosis (1,2) | Concerns about AI's lack of understanding of patients' communication (2,3) | AI faster care  (3, 4) | AI may NOT speed up treatment_journey (1,1) | Would NOT want final decision of AI used in care - up to clinician (1,1) | Clinicians need to know performance of AI (1,2) | Autonomous AI NOT ok (1,1) | Education ‘campaign’ (1,1) |  | No need for human communication of AI result normal – automated (3,5) | AI would raise expectations (2, 7) |
|  | Patients involved in their care  (2,3) |  |  | black box  (7, 8) |  |  | Autonomous AI  (1,1) |  |  |  |  |
|  | AI gives confidence in patient interactions (1,2) | Concern re limitations of the functionality of AI (5,7) | AI for patient healthcare planning (1,1) | AI NOT for symptomatic pt (2,2) | Human oversight needed (6,14) | Public need to know performance of AI (2,3) | Older clinicians stuck in their ways (3, 4) | Education in AI to fulfil professional requirements (1,1) |  | Not liking information about care (patient) (1,1) | Creation of bottle necks at other parts of the system (2,4) |
|  | AI involvement in care as soon as possible (1,1) | HCI more important than ai (3,8) | AI for patient pathway planning (1,2) | Cost of AI (1,1) | Need for careful monitoring of AI_QA (4,6) | Data used to train AI acknowledgement (2,3) | Know nothing about AI  (3, 8) | Education_critical awareness of technology (1,1) |  |  | Need for other parts of healthcare to keep pace with AI diagnosis (2,2) |
|  | Communication for understanding or interest (1,1) |  | AI for patient healthcare planning (1,1) | Data privacy issues (2,2) | AI for second opinion (5,8) | User interface_detail needed (1,2) | AI may not be used by clinicians in the way that it should (1,3) | Clinicians need to see AI implemented to trust it_case based education (1,3) |  |  | Limitations of healthcare system currently - NOT technology (4,11) |
|  | Person to person communication important (12,25) |  | Positivity for AI to help with other issues in relation to condition (1,1) | Need for timing of AI interaction to be optimal (1,2) | Confidence in clinician_diagnosis (1,1) | Variations between asymptomatic and symptomatic patients (6,12) | Humans have skills that AI does not (3,7) | Need for clinical AI specialists (1,3) |  |  | Deskilling of clinicians (3, 3) |
|  | Positive experience with staff in hospital (2,4) |  | AI for screening for disease and patient condition monitoring (5,9) | Patients might be apprehensive about AI in care (1,2) |  |  | Possible issues with those not comfortable with tech - generational issues (1,2) | Need for public education AI SOCIAL MEDIA (1,1) |  |  |  |
|  |  |  | AI gives CONFIDENCE in diagnosis (1,2) | Public overreliance on technology (1.1) |  |  | Potential for over-expectation of AI (1,1) | Positive educational message about AI needed (2,3) |  |  |  |
|  |  |  | AI gives confidence in patient interactions (1,2) | Workflow integration (2,2) |  |  | Variability of clinican acceptance of AI (4,5) | Training_example based (5,9) |  |  |  |
|  |  |  | AI might speed up current systems (11,27) | AI replaces humans  (3, 6) |  |  | Variability of interest of clinicians in AI (1,1) | Public education - fast pace of AI development making training obsolete (1,1) |  |  |  |
|  |  |  | Happy for AI use in care (4,6) |  |  |  | AI resistance (2, 4) | Public lack of use due to lack of understanding (1,1) |  |  |  |
|  |  |  | AI to decrease disparity in care_clinician variability (5,9) |  |  |  | AI acceptance in clinicians is mixed bag (1,1) | Older clinicians “stuck” in their ways (3,4) |  |  |  |
|  |  |  | AI to reduce workload (3,5) |  |  |  | Patient involvement with AI depends on patient personality (2,9) |  |  |  |  |
|  |  |  | AI for triage and pt pathway planning (6,6) |  |  |  | AI resistance(2,4) |  |  |  |  |
|  |  |  | AI for workflow (3,3) |  |  |  | positive usability of AI  (2,5) |  |  |  |  |
|  |  |  | Delay in treatment_current system (2,2) |  |  |  | hearts and minds change perceptions of AI needed  ((3, 6) |  |  |  |  |
|  |  |  | Delayed time to diagnosis w_out AI (2,5) |  |  |  | Know nothing about AI  (3, 8) |  |  |  |  |
|  |  |  | Inevidability of AI involvement in care in future_currently (2,4) |  |  |  | Fear around ~AI  (3,4) |  |  |  |  |
|  |  |  | Positivity for AI to solve specific health problems (1,2) |  |  |  | AI acceptablity in a general sense (3, 7) |  |  |  |  |
|  |  |  | positive change for patients with AI  (2,6) |  |  |  |  |  |  |  |  |
|  |  |  | delayed diagnosis for patients  Without AI (2, 9) |  |  |  |  |  |  |  |  |
|  |  |  | AI positive change  (3,12) |  |  |  |  |  |  |  |  |
|  |  |  | AI positive change , (3 , 12) |  |  |  |  |  |  |  |  |
|  |  |  | AI leads to faster care (3 ,4) |  |  |  |  |  |  |  |  |
|  |  |  | AI speeds up diagnosis clinician perspective (5, 13) |  |  |  |  |  |  |  |  |
